# Centrosome-centric view of asymmetric stem cell division

**DOI:** 10.1098/rsob.200314

**Published:** 2021-01-13

**Authors:** Cuie Chen, Yukiko M. Yamashita

**Affiliations:** 1 Life Sciences Institute, Department of Cell and Developmental Biology, University of Michigan, Ann Arbor, MI, USA; 2 Whitehead Institute for Biomedical Research, Department of Biology, Massachusetts Institute of Technology, Howard Hughes Medical Institute, Cambridge, MA, USA

**Keywords:** centrosomes, stem cell, asymmetric stem cell division

## Abstract

The centrosome is a unique organelle: the semi-conservative nature of its duplication generates an inherent asymmetry between ‘mother’ and ‘daughter’ centrosomes, which differ in their age. This asymmetry has captivated many cell biologists, but its meaning has remained enigmatic. In the last two decades, many stem cell types have been shown to display stereotypical inheritance of either the mother or daughter centrosome. These observations have led to speculation that the mother and daughter centrosomes bear distinct information, contributing to differential cell fates during asymmetric cell divisions. This review summarizes recent progress and discusses how centrosome asymmetry may promote asymmetric fates during stem cell divisions.

## 1. Introduction

Since Boveri described and named it more than 100 years ago [1], the centrosome (so named because it is in the centre of the cell) its has undergone its fair share of ups and downs. Boveri had already made many interesting observations in the early days, including that the centrosome behaves as if it is central to cell division (e.g. it organizes the cell division apparatus, it is abnormal in cancer cells) [2,3], living up to its name. Then, there were twists in more recent years: centrosomes were found to be dispensable for cell division [4,5], and an entire animal (fly) was found to develop without functional centrosomes [6,7]. Despite these twists, a large body of evidence supports that the centrosome plays important roles, mostly through its ability to organize microtubules and cilia [8,9]. Indeed, defects in centrosome number and function have been linked to severe human diseases including ciliopathies and cancer [10].

An emerging area where the centrosome may play critical functions is asymmetric cell division. Asymmetric cell divisions are achieved by the polarization of cells with respect to fate determinants, coupled with spindle orientation [11–13]. As a major microtubule-organizing centre (MTOC) in the cell during interphase and mitosis, the centrosome can have major influences on cell polarity and spindle orientation. Centrosomes within a cell are intrinsically asymmetric, with one centrosome always being older (the mother centrosome) than the other (the daughter centrosome). The mother and daughter centrosome often differ in their MTOC activity (see below). Of note, many stem cell types have been reported to exhibit stereotypical inheritance of the mother or daughter centrosome, leading to a speculation that the centrosome may control asymmetric cell divisions via cell polarization and potentially as a carrier of critical information that can influence the cell fates. With this ‘centrosome-centric’ view, we summarize recent progress in understanding centrosome asymmetries in the context of development, particularly in the context of asymmetric stem cell divisions.

## 2. Centrosome duplication creates intrinsic asymmetries

**Figure 1.**
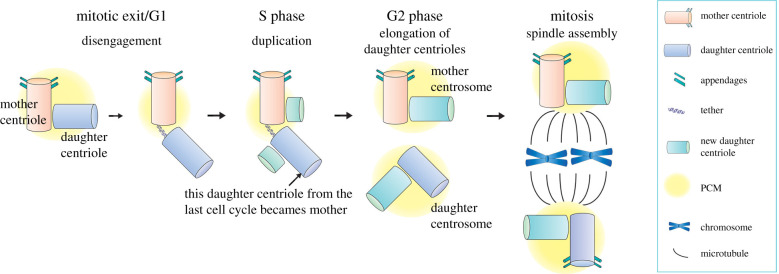
The centrosome duplication cycle in animal cells. At the beginning of the G1 phase of the cell cycle, cells contain a single centrosome that is composed of two centrioles that are orthogonally aligned with each other, surrounded by pericentriolar material (PCM). The mother centriole (orange) served as a template to assemble the daughter centriole (blue) in the previous cell cycle, and can be distinguished from the daughter by its distal and subdistal appendages. Prior to the G1-S transition, the tight juxtaposition of the mother and daughter centrioles is resolved (centriole disengagement) but they remain connected by a fibrous structure called the tether. At the G1-S transition, each centriole initiates the nucleation of new daughter centrioles (green), and the daughter centriole becomes the mother for the first time, but it is not yet mature enough to gain appendages. The new daughter centrioles elongate fully by late G2 phase, and two centrosomes (each containing mother and daughter centrioles) separate from each other prior to mitotic entry. In mitosis, the mother and daughter centrosomes organize the mitotic spindle and segregate into the two daughter cells.

The centrosome is a MTOC in animal cells, and its number per cell is tightly regulated through a precise duplication cycle. Conceptually, its duplication during the cell cycle is similar to that of DNA. A pair of centrioles exists at the core of the centrosome, and these centrioles duplicate using preexisting centrioles as a template (figure 1) [14]. The centriole is a cylindrical barrel-shaped structure that consists of MTs arranged in a nine-fold radial symmetry, the structure remarkably conserved from protists to humans [15,16]. The pair of centrioles are surrounded by the pericentriolar materials (PCM) to help nucleate MTs [17]. Similar to DNA, centrosomes duplicate once per cell cycle in a semi-conservative manner: the pair of centrioles split from each other prior to the G1-S transition of the cell cycle, each serving as a template for generating a new centriole (figure 1) [18]. This process creates two centrosomes, each containing one template centriole and one new centriole.

The semi-conservative nature of centriole duplication creates intrinsic differences in two respects. First, within each centrosome, one centriole (template centriole, called the mother centriole) is older than the other (newly duplicated centriole, called the daughter centriole), creating asymmetry in their age (figure 1). Second, when a cell contains two centrosomes (i.e. two pairs of centrioles) after the duplication, the mother centrioles in each centrosome are not the same age, because one was the template of the other in the previous cell cycle. The different age of the two mother centrioles renders the two centrosomes different from each other: the centrosome that contains the older mother centriole is called the mother centrosome, whereas the one that contains the younger mother centriole (i.e. first time mother) is called the daughter centrosome.

The mother versus daughter centrioles can be distinguished by ultrastructure, function and molecular composition. In mammalian cells, only the mother centriole harbours distal and subdistal appendages (figure 1) and can function as the basal body to assemble cilia, whereas the daughter centriole does not [19]. Subdistal appendages are formed as the centriole matures, and are the major site for MT anchoring. Because it takes more than one cell cycle for the mother centriole to develop these appendages and mature, the mother centrosome, which contains the older mother centriole, typically has a higher MTOC activity than the daughter centrosome, which contains a newly minted mother centriole. This generates functional asymmetry between the mother and daughter centrosomes. Several proteins such as Ninein (Nin), Cep164 and outer dense fibre protein 2 (ODF2) are known to specifically localize to the mother centriole, whereas Centrobin (Cnb) localizes only to the daughter centriole [20–24], creating asymmetries in molecular composition between mother and daughter centrioles. Although centrioles in other species such as *Drosophila* and *C. elegans* do not harbour distal/subdistal appendages as in mammalian cells, the mother centrosomes still exhibit higher MTOC activities than the daughters, suggesting that there is a maturation process that gradually increases the centriole's ability to nucleate/anchor microtubules.

## 3. Asymmetric centrosome inheritance during stem cell divisions

These structural and molecular asymmetries between mother versus daughter centrioles as well as those between mother versus daughter centrosomes fascinated many researchers in the field. Yet, the functional significance of these asymmetries still remains enigmatic. In the last two decades, centrosome asymmetry has been documented in the context of asymmetric stem cell divisions, implying a potential functional significance of centrosome asymmetry.

**Figure 2.**
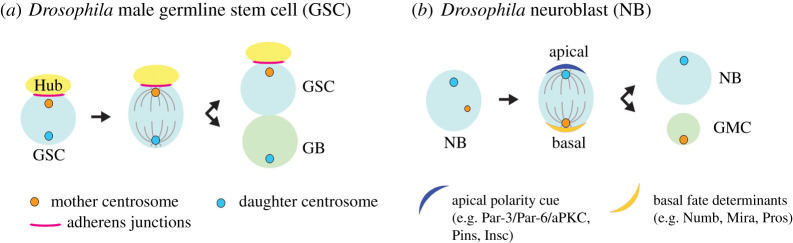
Centrosome inheritance during asymmetric stem cell division. (*a*) *Drosophila* male germline stem cells (GSCs) divide asymmetrically under the influence of signals derived from the hub cells, which function as the stem cell niche. The mother centrosome is anchored at the adherens junctions formed between the GSCs and the hub and orients the GSC mitotic spindle. Upon division, the mother centrosome is always inherited by the GSCs. (*b*) *Drosophila* neuroblasts (NBs) divide asymmetrically by polarizing fate determinants (e.g. Numb, Prospero (Pros) and Miranda (Mira)) at the basal cortex, which are subsequently segregated to differentiating cells (ganglion mother cells). Polarization of these fate determinants and spindle orientation is governed by the apical polarity complexes (e.g. Par3/Par-6/aPKC complex, Pins and Insc). The daughter centrosome is always inherited by the NBs upon division.

Asymmetric stem cell division, observed in many stem cell systems, generates one self-renewing stem cell and one differentiating cell, a key process for tissue homeostasis. This process preserves stem cell number, while generating differentiating cells that compensate for the constant loss of cells in the tissue [11–13]. Several stem cell systems have been reported to exhibit stereotypical centrosome inheritance during asymmetric stem cell divisions, where the mother or daughter centrosome is consistently inherited by stem cells. The first example of asymmetric centrosome inheritance in stem cells was reported in *Drosophila* male germline stem cells (GSCs) (figure 2*a*) [25]. Male GSCs are attached to hub cells, which are post-mitotic somatic cells that provide signalling ligands to neighbouring GSCs to instruct their stem cell identity [26–28]. The physical proximity (direct attachment) to the hub cells determines the stem cell identity of GSCs by allowing them to receive signalling molecules provided by the hub cells [29,30]. Male GSCs divide asymmetrically by orienting their mitotic spindle perpendicularly towards the hub cells such that one daughter cell maintains the attachment to the hub cells and retains the stem cell identity, whereas the other sibling cell initiates differentiation by losing the attachment with the hub cells [31]. This oriented division is achieved by stereotypical centrosome positioning: in GSCs, the mother centrosome stays near the hub cells throughout the cell cycle, whereas the daughter centrosome migrates to the opposite side. As a result, the stem cells always maintain the original mother centrosome through repeated cell divisions. Similarly, the mouse radial glial progenitor cells consistently inherit the mother centrosome during their asymmetric cell division [32], indicating that the stereotypical centrosome behaviour is broadly conserved.

Interestingly, *Drosophila* neuroblasts (NBs) also exhibit a stereotypical centrosome inheritance pattern, but in contrast to *Drosophila* male GSCs and mouse radial glial progenitor cells, they inherit the daughter centrosome (figure 2*b*) [33,34]. NBs are polarized by forming specific cortical domains: the apical side concentrates polarity proteins that regulate spindle orientation, whereas the basal cortical domain recruits factors that specify differentiation [35–38]. The spindle orientation of NBs depends on the Par-3 (Baz)/par-6/aPKC and Pins/Gαi/Mud protein complexes, which form at the apical cortex. Fate-determining factors including Pros, Numb and Mira sit at the opposite side and will be segregated into the ganglion mother cell (GMC), which produces differentiated neurons and glia [35,36]. In this process, the daughter centrosome acquires robust MTOC activity and stays near the apical cortex, whereas the mother centrosome sheds PCM and diminishes the MTOC activity during interphase [33,34,39,40]. Later in the cell cycle, the mother moves to the basal side and regains the MTOC activity right before mitosis. Apart from NBs, *Drosophila* female GSCs also retain the daughter centrosome rather than the mother during asymmetric cell division [41].

In addition to these examples, several other systems exhibit stereotypical centrosome inheritance (table 1). Of note, spindle pole bodies (SPBs), the yeast equivalent of centrosomes, show stereotypical inheritance, where the mother SPB always segregates into bud cells [43], suggesting broad conservation of this phenomenon. Yet, the fact that some stem cell types inherit the mother centrosomes, whereas others inherit the daughter centrosomes, shows that the centrosome age is not directly linked to stemness per se.

**Table 1.** A list of asymmetric centrosome segregation in asymmetric cell divisions.

model	centrosome inheritance pattern	reference
*Drosophila* male GSCs	stem cells inherit the mother centrosome	[25]
*Drosophila* female GSCs	stem cells inherit the daughter centrosome	[41]
*Drosophila* NBs	stem cells inherit the daughter centrosome	[33,34]
mouse neural progenitors	progenitors inherit the mother centrosome	[32]
mouse ES cells	stem cells inherit the mother centrosome	[42]
budding yeast	bud (daughter) cells inherit the old SPB	[43]
human neuroblastoma cells	NuMA+ cell inherits daughter centrosome	[44]

## 4. How could centrosome asymmetry contribute to asymmetric cell fate?

As described above, asymmetric centrosome segregation is obviously conserved through evolution. However, whether and how asymmetric centrosome inheritance may contribute to asymmetric stem cell division remains elusive. Clearly, asymmetric MTOC activities can ensure correct spindle orientation: for example, in *Drosophila* male GSCs, the mother centrosome has higher MTOC activity and is stably anchored to the adherens junctions formed between the hub and GSCs (figure 2*a*), ensuring that a spindle pole is tethered at the hub, which in turn leads to perpendicular spindle orientation in mitosis [25]. In this scenario, maintaining the mother centrosome in stem cells may be nothing more than ‘convenience’ and just a byproduct of anchoring the spindle pole.

However, much more elaborate cellular mechanisms of asymmetric centrosome inheritance in *Drosophila* NBs suggest that the story might not be that simple. As mentioned above, NBs inherit the daughter centrosome [33,34] because the newer, daughter centrosome acquires a strong MTOC activity, whereas the mother sheds PCM to become inactive. Multiple mechanisms contribute to creating the asymmetry between the mother and daughter centrosomes in *Drosophila* neuroblasts. The daughter centrosome's MTOC activity is upregulated by recruitment of Cnb and Polo, which occurs during mitosis in preparation for centrosome asymmetry in the next interphase [45,46]. In parallel, the mother centrosome's MTOC activity is downregulated, releasing it from the apical cortex, leading to its eventual inheritance by the differentiating cell. The downregulation of the mother centrosome's MTOC activity requires Bld10/Cep135 and Plp, and mutations in these genes result in two active centrosomes, leading to randomized inheritance of the centrosomes [47,48]. It is difficult to explain this phenomenon, the elaborate switching MTOC activities between two centrosomes, solely on the basis of needing to anchor one centrosome. Based on these observations, it is natural to speculate that the centrosome asymmetries may have additional roles that lead to asymmetric cell divisions.

How could asymmetric cell fates arise from asymmetries between mother and daughter centrosomes? Several possibilities have emerged to explain how asymmetric mother–daughter centrosomes can drive asymmetric cell division, as summarized below.

### 4.1. Association with fate determinants

**Figure 3.**
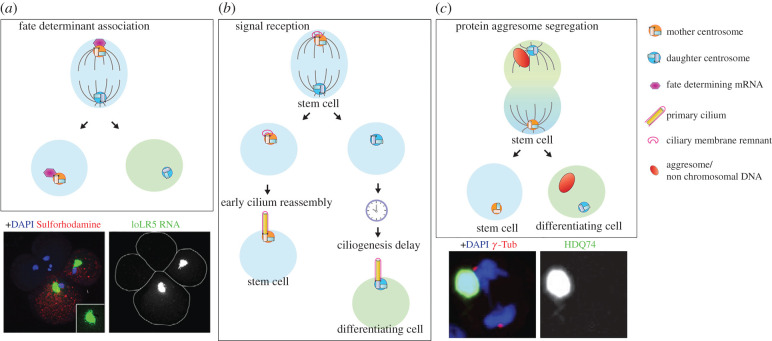
Function of centrosome asymmetry in asymmetric cell division. (*a*) During early embryonic development of the mollusc IIyanassa, fate-determining mRNAs associate with the centrosome and segregate asymmetrically to one of two daughter cells. Lower panel: An example of asymmetric segregation of mRNAs. Image adapted from [49]. (*b*) During mouse brain development, centrosome asymmetry leads to a biased reception of signals between two daughter cells in asymmetrically dividing apical progenitors (APs). The mother centrosome is associated with the ciliary membrane remnant, allowing the cell to reassemble the cilia quickly upon mitotic exit and to retain the stem cell character. The sibling cell that inherited the daughter centrosome, which lacks the remnant ciliary membrane, takes longer to build the cilium and enters the differentiation programme. (*c*) The aggresome is associated with one centrosome and segregates asymmetrically during mitosis in multiple mammalian cell types including human embryonic stem cells (ESCs). Lower panel: An example of asymmetric segregation of the aggresome. Image adapted from [50].

Asymmetric centrosome inheritance may be linked to the segregation of fate determinants (figure 3*a*). An elegant study has illustrated that fate-determining mRNAs are associated with one centrosome during cell divisions of the mollusc embryo, governing binary fate decision [51]. During early cleavage cycles of embryonic development, distinct mRNAs (*IoDpp*, *IoEve* and *IoTld*) are associated with one of the two centrosomes, and segregated to only one daughter cell. The centrosome-localized mRNAs accumulate in specific cells via asymmetric segregation, giving rise to embryonic patterning during mollusc development. However, in this case, it remains unclear whether the association with fate determinants is linked to centrosome age (mother versus daughter). More recently, a regulator of Notch ligand activity, Mindbomb1 (Mib1), was found to localize asymmetrically to the daughter centrioles in chick neural progenitors, leading to its segregation to prospective neurons during mitosis [52]. Disruption of such biased Mib1 localization leads to symmetric divisions, and eventually a reduction in neurogenesis, showing that asymmetric segregation of fate determinants (e.g. Mib1) is achieved by their association with the centrosomes.

### 4.2. Differential signal reception through primary cilia assembly

Another intriguing possibility by which centrosome asymmetry may contribute to asymmetric cell fates is through a differential ability of the mother versus daughter centrosome to assemble primary cilia. Upon completion of mitosis in cultured mouse NIH 3T3 fibroblast cells, the cell that inherits the mother centrosome grows the primary cilium earlier than its sibling and is, as a consequence, briefly more sensitive to Sonic hedgehog (Shh) signalling [53]. In mouse radial glial progenitor cells, the mother centrosome does not completely disassemble the primary cilium when cells enter mitosis, and the remnant of the ciliary membrane is attached to the mother centrosome throughout mitosis, serving as a seed to reassemble primary cilia upon mitotic exit (figure 3*b*) [54]. This observation may explain why the mother centrosome-containing cells assemble primary cilia faster than their siblings [53]. As a result, the cells that inherit the mother centrosome accumulate more Smoothened (Smo) and experience higher hedgehog (Hh) signalling, which promote stem cell identity. On the other hand, their siblings that inherit the daughter centrosome do not self-renew due to lower Hh signalling and commit to differentiation [54].

These studies revealed an elegant mechanism by which a subtle difference between two sibling cells, such as centrosomal age, may be amplified to instruct their distinct cell fates. Even if cell division is originally ‘symmetric’ (i.e. without asymmetric fate determinants), the differential timing of cilia formation can act as a symmetry-breaking event, making two daughter cells distinct from each other, despite their similarity at birth.

### 4.3. Asymmetry in cells' age

Other intriguing examples of asymmetric cell division are a broadly observed biased segregation of ‘aging factors’, including aggresomes and non-chromosomal DNA, such that certain cells (e.g. stem cells) can avoid/delay aging.

The aggresome, a large aggregate of damaged/misfolded proteins, was found to be associated with one centrosome during division, leading to its asymmetric inheritance: cells typically form a large, single aggresome per cell, and consequently, its centrosome association during mitosis leads to one daughter cell with the aggresome and the other without (figure 3*c*) [50,55,56]. During human ES cell divisions, aggresomes were found to be preferentially inherited by the non-stem daughter [55]. However, it was not clear from these studies whether the aggresome was consistently associated with the mother or daughter centrosome. It has been speculated that this asymmetric segregation of the aggresome to differentiating daughter cells may help to extend the lifetime of stem cells.

Similarly, non-chromosomal DNA, such as extra chromosomal circles (ERC) generated by intrachromatid recombination of repetitive DNA (e.g. rDNA repeats), is segregated asymmetrically to mother cells during yeast cell divisions [57]. Accumulation of ERCs has been linked to replicative senescence [58,59], and therefore asymmetric partitioning of ERCs to the mother cells serves to preserve the lifespan of the daughter cells. The age of SPBs is indeed a critical determinant of asymmetric ERC partitioning [60], revealing the importance of centrosome/SPB and their mother/daughter asymmetry in governing asymmetric segregation of aging factors. A recent study showed that foreign DNA (due to plasmid transfection) is segregated asymmetrically by preferentially associating with the daughter centrosomes [61], suggesting that the asymmetric segregation of non-chromosomal DNA may be a broadly employed mechanism to protect cells.

Although its functional significance remains unknown, midbody inheritance has been connected to centrosome age. The midbody is the structure that is left behind upon completion of cytokinesis, which is composed of the remnant of the contractile ring and central spindle MTs [62]. Since the midbody cannot be split in half, it is inherited by one of the two daughter cells. Although the midbody is not physically associated with centrosomes, a strong correlation between midbody inheritance and centrosome age has been documented. In HeLa cells, it was shown that the midbody goes to the cell that inherits the mother centrosome [63]. In addition, an interesting correlation was observed between midbody inheritance and cell fate: stem cells and cancer cells were observed to inherit and accumulate midbodies, whereas cells release midbodies upon the induction to differentiate [64,65]. In dividing *Drosophila* male and female GSCs, the midbody was inherited by the daughter centrosome-containing cells (i.e. stem cells in the female germline and differentiating cells in the male germline) [41]. Recently, lysosomes were reported to concentrate near one centrosome of keratinocytes and were preferentially inherited by a daughter cell that yields colonies expressing the stem cell marker KRT15 [66].

Although the functional significance of asymmetric segregation of these cellular organelles/components is not always clear, the centrosomes often regulate their segregation patterns, therefore, centrosomes appear to be in an ideal, ‘central’ position to govern and orchestrate the segregation of multiple organelles and other cellular components. Just like a master transcription factor that regulates cell fate by controlling many downstream targets, the centrosome may regulate cell fate by governing many downstream events.

## 5. Do centrosome proteins reveal how centrosomes could contribute to asymmetric fates?

Despite mounting examples of asymmetric behaviours for mother and daughter centrosomes during stem cell divisions, direct evidence that such asymmetries contribute to asymmetric cell fate is still lacking. This is primarily because it has been difficult/impossible to perturb centrosome asymmetry without perturbing other aspects of centrosome functions. Specifically targeting centrosome asymmetry probably requires genes/factors that only regulate centrosome asymmetry. Once such factors are identified, it may be possible to perturb centrosome asymmetries in stem cells to examine the consequences. In recent years, several centrosomal proteins that exhibit enrichment in the stem cell centrosomes have been identified. Although none of them gave the direct answer on the ‘functional relevance of centrosome asymmetry’, these studies have added confidence to the notion that centrosome asymmetry must be a critical aspect of asymmetric stem cell division. Future studies that investigate the function of these proteins may provide deeper insights into the role of centrosome asymmetry in asymmetric stem cell divisions.

### 5.1. Klp10A

**Figure 4.**
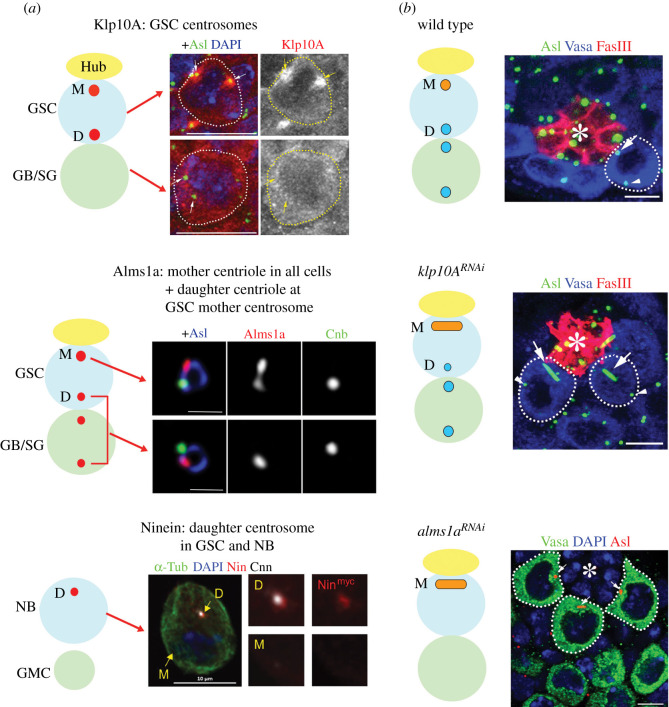
Proteins that exhibit stem cell-specific centrosomal localization and their functions. (*a*) Protein localization of Klp10A, Alms1a and Ninein in *Drosophila* stem cells. Images from [68–70]. (*b*) Phenotypes upon knockdown of *klp10A* and *alms1a*. *klp10A* depletion leads to elongation of the mother centrosomes in *Drosophila* male GSCs. Depletion of alms1a leads to a loss of the daughter centrosomes and elongation of the mother centrosome. Asl marks centrosomes, Vasa germ cells and FasIII hub cells (also marked by asterisks).

Klp10A is a microtubule-depolymerizing kinesin of the kinesin-13 family [67], and was identified as the first stem cell-specific centrosomal protein [68]. It localizes to the stem cell centrosomes, but not the centrosomes of differentiating germ cells in the *Drosophila* male germline (figure 4*a*) [68]. Depletion of Klp10A resulted in an abnormally elongated mother centrosome, without affecting other centrosomes (GSC daughter centrosome and any centrosomes of differentiating cells), revealing a unique regulation imposed on the GSC mother centrosome (figure 4*b*). The long mother centrosome and normal daughter centrosome in GSCs results in aberrant asymmetries during GSC division, i.e. a mitotic spindle with a large and a small half spindles, leading to asymmetric daughter cell sizes (a bigger GSC and a smaller differentiating gonialblast (GB). The small GBs frequently die, possibly due to insufficient cellular contents for viability. Although these results do not uncover the meaning of centrosome asymmetry, they imply that centrosome asymmetry may arise from an intricate balance between the forces that generate centrosome asymmetry and the forces that counteract it. The elongation of the mother centrosome upon *klp10A* depletion suggests the presence of a mechanism that continuously elongates the mother centrosome, hence a unique mechanism imposed on the mother centrosome, unless counterbalanced by *klp10A*. It remains elusive what is being counteracted by Klp10A.

### 5.2. Alms1a

Recently, Alms1a, a *Drosophila* homologue of the causative gene for human ciliopathy Alstrom syndrome [71,72], was identified as a GSC-specific Klp10A-interactor [69]. Alms1a was found to be a pan-mother centriole protein, but exhibits additional localization to the daughter centriole specifically in the mother centrosome of *Drosophila* male GSCs (figure 4*a*) [69]. Strikingly, upon knockdown of *alms1a,* GSCs failed to duplicate their centrioles, leading to centrosome loss in all of their progeny, while the original mother centriole within GSCs continued to elongate (figure 4*b*). Another striking feature of *alms1a* function is that it is required for centriole duplication only in asymmetrically dividing GSCs, but not in symmetrically dividing GSCs nor differentiating cells. Alms1a promotes centriole duplication probably via its interaction with Sak, the *Drosophila* homologue of Plk4 kinase, a master regulator of centriole duplication [73,74]. These data again demonstrate a unique characteristic of the GSC mother centrosome. However, it remains unanswered why the stem cell centrosomes are asymmetric and different from centrosomes in non-stem cells.

### 5.3. Ninein

Ninein is a protein enriched on the mother centriole [23]. Mutations in Ninein cause human Seckel syndrome [75]. In mouse radial glial progenitor cells, Ninein was found to be enriched on the mother centrosome and inherited by the radial glial progenitor cells upon their asymmetric division. Moreover, Ninein is required for the stereotypical inheritance of the mother centrosome by these progenitor cells [32]. In Drosophila, Ninein was also found to be enriched on the mother centrosomes in NBs and male GSCs (figure 4*a*) [70]. Despite such an intriguing localization, depletion of Ninein does not detectably impact stem cell divisions or fates in Drosophila stem cells, therefore, the relevance of its localization remains unknown.

Whereas Ninein is consistently associated with the mother centrosome in these cell types, it does not appear to correlate with cell fate or MTOC activity. The Ninein-enriched mother centrosome is inherited by the stem cells in mouse radial glial progenitors and *Drosophila* male GSCs, whereas it is inherited by the differentiating daughters upon *Drosophila* NB division. Likewise, whereas the Ninein-enriched mother centrosomes have robust MTOC activity in mouse radial glial progenitors and *Drosophila* male GSCs, they have downregulated MTOC activity in *Drosophila* NB. Thus, it remains unclear how Ninein may contribute to asymmetric stem cell divisions.

Altogether, these studies have finally began to identify stem cell-enriched centrosomal proteins, and their phenotypes reveal the necessity of regulating stem cell centrosomes. However, we are still left wondering whether asymmetries between the mother and daughter centrosomes have functions beyond their ability to organize MTs and orient stem cell divisions.

## 6. Conclusion and outlook

Asymmetric stem cell division is fundamental to tissue homeostasis, and it is a robust and complicated process that requires multiple layers of control. The asymmetric behaviours of the mother and daughter centrosomes can be used to control asymmetric cell division and assist the specific needs of various stem cells during development and differentiation. Here, we summarized the current knowledge in this area, highlighting the evidence that centrosome asymmetry does contribute to asymmetric fate determination.

Although there are individual examples of fate-determining factors associating with the centrosomes, we still lack a comprehensive understanding of how centrosomes can generally contribute to asymmetric cell division. Ultimately, to determine the centrosome's role in asymmetric cell division, we must experimentally abolish centrosome asymmetries and explore the functional outcome.

## Data accessibility

This article has no additional data.
